# Case Report: Suppression of Harem Stallion Behavior and Fertility Following Anti-Gonadotropin-Releasing Hormone Vaccination of a Captive Wild Przewalski's Horse (*Equus ferus przewalskii)*

**DOI:** 10.3389/fvets.2020.569185

**Published:** 2020-11-24

**Authors:** Jérôme Ponthier, Goulven Rigaux, Sonia Parrilla-Hernandez, Sophie Egyptien, Carine Gatez, Carla Carrasco Leroy, Stéfan Deleuze

**Affiliations:** ^1^Equine and Companion Animal Reproduction Pathologies Clinic, Veterinary Medicine Faculty, University of Liège, Liège, Belgium; ^2^Domaine des Grottes de Han, Han sur Lesse, Rochefort, Belgium; ^3^Animal Physiology, Veterinary Medicine Faculty, University of Liège, Liège, Belgium

**Keywords:** wildlife preservation, Przewalski horse (*Equus ferus przewalskii*), anti-GnRH immunization, neutering, castration, electro-ejaculation

## Abstract

This report describes an option to modulate the testicular function of wild horses and field methods to assess it. Non-surgical castration of a captive wild Przewalski's stallion with anti-gonadotropin-releasing hormone (GnRH) immunization was performed by sub-cutaneous injection of two doses of 450 μg (3 ml) of GnRH conjugated to diphtheria toxin, further repeated every 6 months. Semen quality was assessed after collection by electro-ejaculation under general anesthesia. Endocrine and behavioral consequences were studied during a 2-year follow-up period. The procedure of electro-ejaculation was safe and effective to collect spermatozoa. Motility was low but was improved by a significant dilution of sample (1*v*/4*v*−1*v*/5*v*) after collection. Immuno-neutering resulted in a decrease of the total spermatozoa number and motility 1 month after primary vaccination. However, infertility could not yet be guaranteed. Six months post-vaccination, serum testosterone concentrations had decreased and the treated stallion had lost his harem stallion role. Moreover, at the same time, the total spermatozoa number was near zero with no motile spermatozoa, and offspring was no longer observed. As a conclusion, electro-ejaculation under general anesthesia is suitable on wild horses to obtain spermatozoa that should be washed or largely diluted before use for artificial insemination (AI) programs. Anti-GnRH immuno-neutering protocol led to a dramatic decrease of spermatozoa number, motility, and testosterone production. This also induced deep changes in the social structure of the band. Such technique could be considered as an alternative to surgical castration in wild horses.

## Introduction

In zoos and safari parks, modulation of reproduction is required to limit the offspring of some over-represented species or inbreeding within herds. However, neutering of captive wild animals is challenging: any intervention is time consuming and life threatening when a general anesthesia is required. Moreover, any surgery is including risks of wound infection that would be difficult to treat.

Modulation of the equine hypothalamic–pituitary axis can be an alternative to surgical neutering (1). Vaccination against gonadotropin-releasing hormone (GnRH) induces a decrease of testosterone and spermatozoa production in domestic stallions, and commercial contraception protocols are available (2). The main adverse effects are a local inflammation after injection and a persistent infertility in males or anestrus in mares, sometimes lasting long after the last vaccination (2, 3). Inhibition of testosterone secretion interferes with the social behavior in gregarious species (4–8). In wild horses, the harem stallion may be challenged by other males, and rapid changes in hierarchy may cause severe injuries after fights (9, 10).

This report aims to describe the effects of an anti-GnRH vaccination in a captive wild Przewalski's horse (*Equus ferus przewalskii*) on semen quality, testosterone concentrations, and social interactions over a long period.

## Case Description

**Figure 1:**
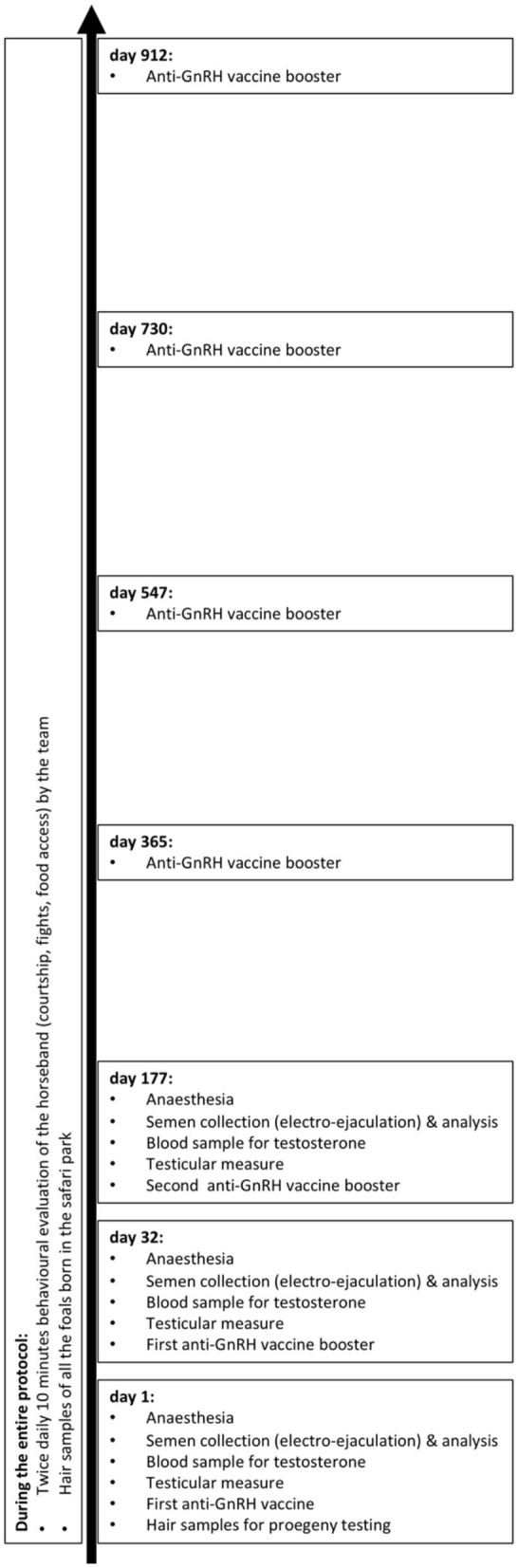
Timeline of the procedures and observations realized on the treated stallion and the Przewalski's horseband.

“Le Domaine des Grottes de Han” (Han sur Lesse, Belgium) is a safari park housing Przewalski's horses roaming freely. Depending on the season, hay supplementation is distributed once or twice a day in the only feeder available in the park. Castration of the 18-year-old harem role stallion was considered to limit the band's inbreeding, as he was keeping two bachelors away from the three or four mares, their foals (one or two, depending on the year), and the food. The immuno-neutering with an anti-GnRH vaccine was preferred over surgical procedures. The vaccine consisted in a 3-ml dose of Improvac® (vaccine initially developed for porcine by Zoetis, Louvain-la-Neuve, Belgium) containing a minimum of 450 μg of GnRH agonist conjugated to diphtheria toxin, as specified by the manufacturer leaflet. On day 0 (May 26th 2016), day 32, and day 177, the vaccine was subcutaneously administrated in the chest, during the procedures performed under general anesthesia to assess the effects of immuno-neutering on semen quality and testicular endocrine activity with semen and blood samples (Figure 1). Then, for a 2-year period, 3-ml boosters were darted every 6 months. Social behavior with younger stallions and reproductive interactions with mares of the treated stallion were observed twice daily. The treated stallion's paternity was genetically excluded in foals born during these 2 years (Figure 1).

### Anesthesia

The stallion's bodyweight was estimated at 300 kg. The protocol consisted of a tele-anesthesia with a pressurized air dart containing detomidine (10 mg), butorphanol (12 mg), and etorphine (5.6 mg). Once in a recumbent position, the premedication dart was removed, a catheter (14G) was placed in the jugular vein, and intravenous balanced anesthesia (glycerol guaiacolate 500 ml, ketamine 1 g, and xylazine 250 mg) was given on demand until the end of procedures. Oxygen was given via an endotracheal tube. When the procedures were completed, naltrexone (125 mg) and atipamezole (between 25 and 50 mg) were injected intra-muscularly to accelerate recovery.

### Breeding Soundness Examination, Semen Collection, and Analysis

At days 0, 32, and 177, the testicular transverse circumference was measured perpendicularly to the testis long axis with a tape: with three measures of each testis, the mean value was calculated. To decrease the urinary contamination of semen, the bladder was emptied by catheterization before electro-ejaculation. The prostate was manually stimulated during 2 min by rectal palpation.

Ejaculation was induced with an electro-ejaculator using a lubricated bovine rectal probe (Minitübe, Tiefenbach, Germany, 40-cm long, 7-cm diameter, three longitudinal electrodes). Four cycles of stimulations separated by 3 min of rest were performed, with these sequences:

- Cycle 1: 10 stimulations 0.5 V−10 stimulations 1.5 V−15 stimulations 2.5 V;
- Cycle 2: 10 stimulations 0.5 V−10 stimulations 1.5 V−10 stimulations 2.5 V−15 stimulations 3.5 V;
- Cycle 3: 10 stimulations 1.5 V−10 stimulations 2.5 V−10 stimulations 3.5 V−15 stimulations 4.5 V;
- Cycle 4: 10 stimulations 0.5 V−10 stimulations 2.5 V−10 stimulations 3.5 V−15 stimulations 4.5 V.

Semen samples were collected after each cycle. After the last cycle, the bladder was catheterized and the urine was centrifuged to determine the spermatozoa concentration in the pellet.

The following analysis were performed on each semen sample. In the field, immediately after collection, subjective evaluation of spermatozoa motility was performed under light microscopy (×400), with 10 μl of semen on a warmed slide (37°C). To determine the concentration of spermatozoa, 10 μl of semen were diluted in 390 μl of 40% formaldehyde in the field. This sample was then shipped to the laboratory where concentration was determined under microscopy, using a Thoma cell (IMV, Laigle, France). Fractions of the ejaculate were then largely (1*v*/4*v*−1*v*/5*v*) extended with INRA 96® (IMV, Laigle, France) to dilute possible urine contamination of the semen. It was shipped within 4 h to the laboratory, where total and progressive motilities were determined (11) with a Makler® cell in a semen analyzer (IVOS with 2006 software, Hamilton Thorn®, Beverly, USA) using semen re-diluted to 20 × 10^6^ spz/ml with INRA 96® (IMV, Laigle, France). Percent of morphologically abnormal spermatozoa was determined by counting 200 spermatozoa after Diff-Quick® staining (RAL, Martillac, France).

### Endocrinology

Blood samples were collected on days 0, 32, and 177 and assayed for testosterone with an automated spectrography by a commercial laboratory (LabForVet, Les Isnes, Belgium).

### Behavioral Evaluation

Since the beginning of the protocol (day 0), and for 2.5 years, the animal nursing team managers of the safari park recorded interactions between the old treated Przewalski's stallion and the rest of the horse band during the twice-daily 10-min surveillance duty (early morning and afternoon): fights, fights for access to hay in feeders, and aggressions when the treated male was approaching the rest of the group. Courtship behaviors expressed by the males of the band were also reported twice daily in a standardized form.

### Progeny Testing

An equine commercial progeny test was used to determine if the foals born in the park between 2017 and 2019 were the offspring of the treated stallion. These assays used 13 DNA markers, and filiation was excluded when more than five markers differed (Progenus, Gembloux, Belgium).

## Results

### Semen Quality

Table 1 summarizes the volume and semen parameters of four fractions collected by electro-ejaculation on day 0. Repetition of cycles was associated with decreased concentrations and motility in the samples. Few spermatozoa were observed in urine. The total semen volume was 133 ml and the total spermatozoa number was 4,110 × 10^6^ spz. The morphology analysis showed 82.6% of normal spermatozoa and 11.4% of broken mid-pieces. Left and right testis circumferences were 13.5 and 12.5 cm, respectively.

**Table 1:** Evolution of semen parameters.

	**Volume (ml)**	**Spermatozoa concentration (×10^**6**^ spz/ml)**	**Total spermatozoa number (×10^**6**^ spz)**	**CASA total motility after dilution in INRA 96^®^ (%)**	**CASA progressive motility after dilution in INRA 96^®^ (%)**
**DAY 0 SEMEN ANALYSIS**					
Cycle 1	30	65	1,950	89	70
Cycle 2	52	28	1,456	70	48
Cycle 3	22	18	396	76	62
Cycle 4	28	11	308	59	45
**Total**	**132**		**4,110**		
*Bladder*	*181*	*1*	*181*		
**DAY 32 SEMEN ANALYSIS**					
Cycle 1	12	31	372	47	29
Cycle 2	15	48	720	61	37
Cycle 3	17	25	425	41	25
**Total**	**44**		**1,517**		
**DAY 177 SEMEN ANALYSIS**					
Cycle 1	12			No cellular liquid	
Cycle 2	15	<0.0033		No motile cells	
Cycle 3	10			No cellular liquid	
**Total**	**37**		**49,500**		

At day 32, only three cycles of electro-ejaculation were performed, due to anesthesia instability. Data about collected fractions are summarized in Table 1. The total collected volume was 44 ml, with a total spermatozoa number of 1,517 × 10^6^ spz. Day 32 motility parameters were reduced and morphological analysis only showed 58.7% of normal spermatozoa, with high proportions of abnormal mid-pieces (32.5%). No spermatozoa were observed in the urine. The mean testicular circumference was 11 cm for both testes.

**Figure 2:**
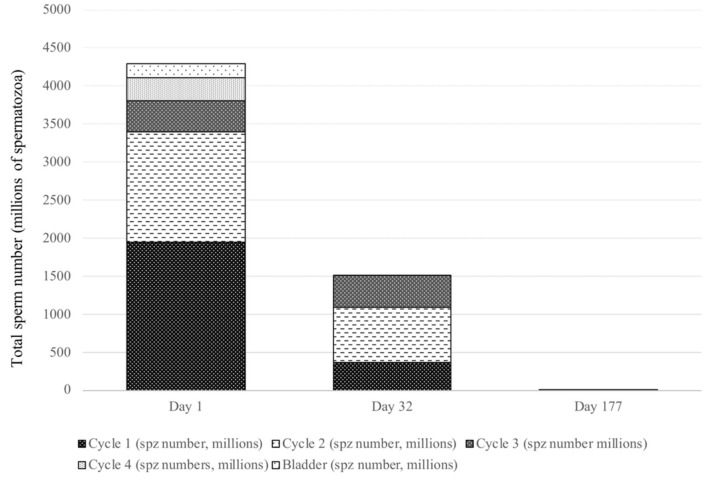
Immuno-neutering effect on stallion's number of collected spermatozoa.

**Figure 3:**
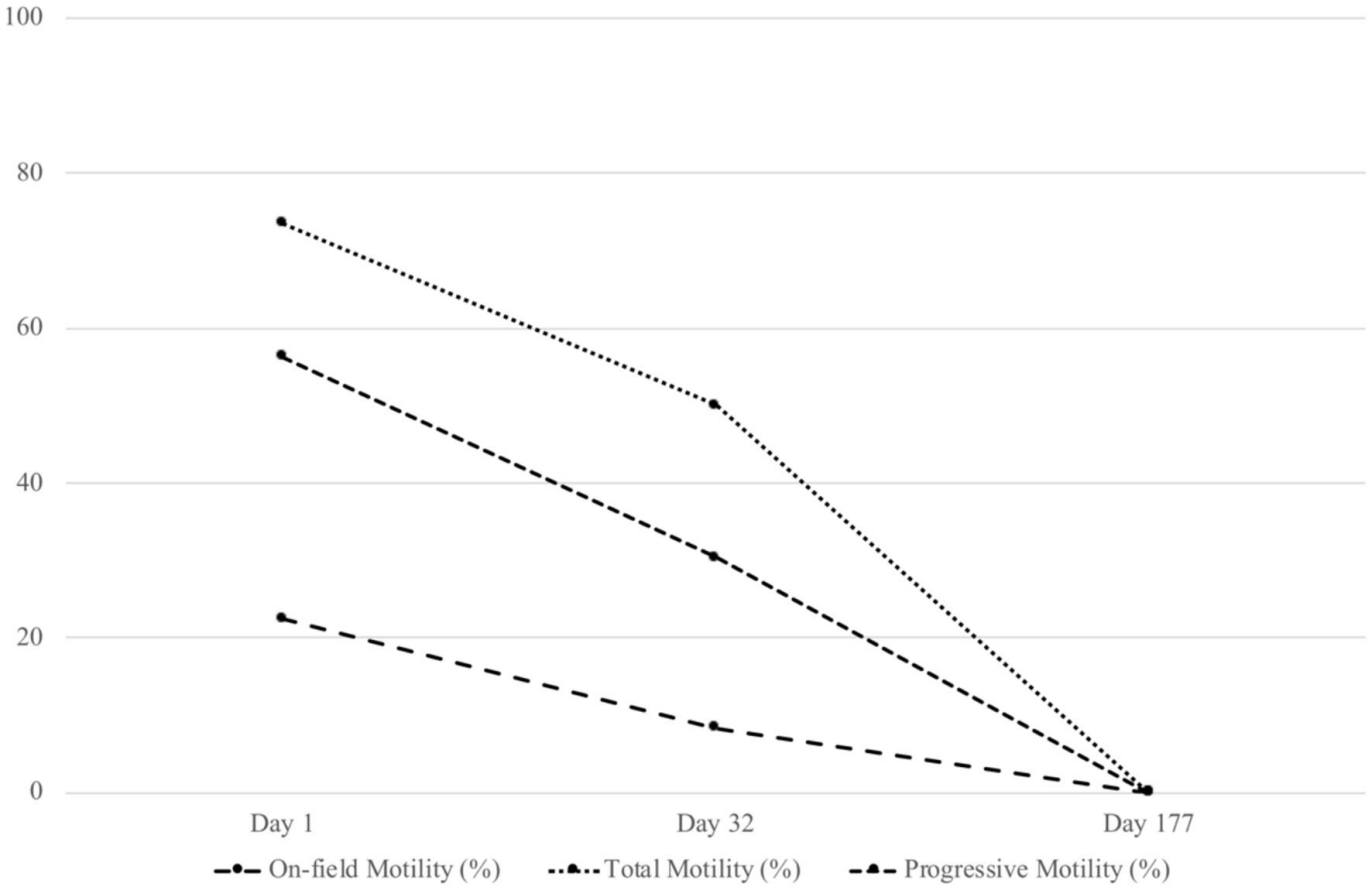
Immuno-neutering effect on stallion's on-field observed motility, total (CASA), and progressive motility (CASA).

At day 177, the total collected volume of semen was 37 ml, with no spermatozoa under light microscopy. Fractions were centrifuged (1,000 × *g*, 20 min), to obtain 1-ml pellets for analysis: 10 μl of these undiluted samples were injected in the Thoma cell, using a correction factor to calculate the concentration. The first and third fractions contained no spermatozoa and the second fraction (15 ml) contained very few (Table 1). A dramatic decrease in the total number of collected spermatozoa was observed, with only 49,500 spz, and no observed spermatozoa was motile (Figures 2, 3). Normal spermatozoa were absent (main abnormalities were mid-piece defects and separated heads). The left testis circumference was 10 cm. The right testis seemed smaller, but could not be measured because it was high-up in the inguinal region.

Between 2017 and 2019, no offspring of the treated stallion was genetically identified in the park.

### Endocrine and Behavioral Evolution

On days 0 and 32, basal blood testosterone concentrations were 105 and 133 ng/dl, respectively. On day 177, it dropped to 29 ng/dl.

Between days 0 and day 32, no behavioral changes were observed: the treated stallion remained the harem stallion, and its body condition was good. The younger stallions were not allowed to eat at the same time or to mate. Between the first and the second booster injections (days 32 and 177), clear changes in the males' hierarchy appeared. Within 2–4 weeks, the treated stallion no longer fought for the leadership and no longer covered the mares. The younger stallions kept him away from the females and the entire band during feeding. His body condition decreased (ribs easy to see, reduced back muscles thickness), reducing his chances to keep the leadership.

After day 177, the anti-GnRH vaccine (3 ml) was darted every 6 months without necessitating a general anesthesia. During these 2 years, the old stallion did not fight to keep his harem stallion role and remained isolated from the band, especially during feeding times. However, 1–2 weeks before the planned time for the booster, the treated stallion showed feces marking and made attempts to fight the leaders, but the vaccine rapidly (2–3 days) suppressed these behaviors.

## Discussion

Our electro-ejaculation protocol under general anesthesia is suitable for semen evaluation and artificial insemination (AI) programs of captive wild Przewalski's horses and, potentially, of other wild equids. As an example, electro-ejaculations were concomitantly performed to include other Przewalski's stallions in an exchange program: motile spermatozoa were also collected (12). Analysis performed before our immuno-neutering protocol showed a low raw semen quality, but the large dilution (1*v*/4*v*−1*v*/5*v*) with a commercial extender improved motility. This reversible effect was also observed by our team (12) and in another report of wild Przewalski's horses electro-ejaculations (13). This could be explained by the dilution of urine contamination: further studies should assay urea and creatinine in raw semen and correlate it with the semen quality. However, the motility improvement observed after dilution suggested that electro-ejaculated spermatozoa of wild equids are suitable for AI after washing with two or three centrifugations in an extender, as described for toxic plasma (14).

The first three doses of anti-GnRH vaccines were given during anesthesia, which was needed to monitor the evolution of semen quality of this wild stallion. Tele-injection was then easily performed every 6 months on this stallion because the vaccine volume was low (3 ml). In agreement with the previous reports on equine domestic breeds, total azoospermia and a nearly complete aspermia were observed after 6 months (1–3, 15–17), when this can require up to 1 year in elephants (7, 8).

Basal blood testosterone concentrations showed a dramatic decrease between days 32 and 177. No dynamic exploration of testosterone production after an hCG stimulation was performed due to short-time general anesthesia performed in the field. Thus, the low testosterone concentration observed on day 177 could only be a temporary decrease between the 4 to 6 peaks daily observed in stallions (18–21). However, the dramatic decrease in testicular size, spermatozoa number and quality, and the absence of offspring during the follow-up all confirm the reduced testicular functions after an anti-GnRH immuno-neutering.

The treated stallion's behavior also highly suggested a decreased testosterone production. Changes in hierarchy were observed after the first booster injection (day 32), leading to the loss of harem stallion role by the immuno-neutered animal. This effect was already documented in wolves, which are also living in complex social structures (4, 5). In wild equids bands, immuno-neutering affects not only the treated stallion's interactions with other horses but also his feeding behavior and body condition. This could be related to the new harem stallions' roles forbidding the old one to access to the feeders or to the observed testosterone decrease. Resumption of fights between the new harem stallions' roles and the old one before the booster injection supports a rapid reversibility of the vaccination effect.

Surgical castration or immuno-neutering have behavioral effects that should be compared to the risk and benefits of a surgical vasectomy. In the present case, avoiding inbreeding was getting urgent and this old stallion could not be included in an exchange program. As euthanasia was not an option for the park team, they were informed about each option's risks. Castration or vasectomy post-operative cares seemed impossible to perform for the team on a free roaming wild horse, and immuno-neutering was chosen as the best solution. The staff still manages the consequences for the treated stallion by feeding him separately.

The effects of immuno-neutering are well-described in stallions (1–3). This non-surgical option is an effective, easy, safe, cheap, and possibly reversible means to control wild horse populations and only requires darting every 6 months. It could improve animal welfare by avoiding euthanasia when control of the equid's population is becoming imperative: not only in parks but also where free groups of wild horses, wild ass, donkeys, or zebra cause troubles to their environment. However, reproduction data in the larger bands of immuno-neutered stallions and mares could help to understand the long-term effects of this technique.

To conclude, the electro-ejaculation protocol performed under general anesthesia was effective to collect spermatozoa for semen quality assessment of a captive wild Przewalski's horse. Dilution and/or washing of the raw semen could increase its quality for fresh semen AI procedures. Vaccination against GnRH resulted in an efficient fertility suppression in this Przewalski's stallion and induced anatomical, endocrine, and behavioral changes, contributing to the loss of his harem stallion role. Such technique should always be considered before performing any kind of castration in groups of wild animals.

## Data Availability Statement

The raw data supporting the conclusions of this article will be made available by the authors, without undue reservation.

## Ethics Statement

Ethical review and approval was not required for the animal study because the procedures were performed on an animal on the demand of the owner (a safari park). The study was realized on the basis of this clinical situation, required by the owner of the animal. Written informed consent was obtained from the owners for the participation of their animals in this study.

## Author Contributions

All authors performed all the on-field procedures, including anesthesia, and electro-ejaculation. JP, SP-H, SE, and SD performed the semen analysis. GR and CCL collected the comportemental information during all the follow-up period. They also darted the stallion for booster vaccines after day 177. JP wrote the manuscript. All authors reviewed and corrected the manuscript. All authors contributed to the article and approved the submitted version.

## Conflict of Interest

The authors declare that the research was conducted in the absence of any commercial or financial relationships that could be construed as a potential conflict of interest.
